# Loss of Intralipid®- but Not Sevoflurane-Mediated Cardioprotection in Early Type-2 Diabetic Hearts of Fructose-Fed Rats: Importance of ROS Signaling

**DOI:** 10.1371/journal.pone.0104971

**Published:** 2014-08-15

**Authors:** Phing-How Lou, Eliana Lucchinetti, Liyan Zhang, Andreas Affolter, Manoj Gandhi, Martin Hersberger, Blair E. Warren, Hélène Lemieux, Hany F. Sobhi, Alexander S. Clanachan, Michael Zaugg

**Affiliations:** 1 Cardiovascular Research Centre, University of Alberta, Edmonton, Alberta, Canada; 2 Department of Anesthesiology & Pain Medicine, University of Alberta, Edmonton, Alberta, Canada; 3 Department of Clinical Chemistry, University Children's Hospital Zurich, Zurich, Switzerland; 4 Department of Pharmacology, University of Alberta, Edmonton, Alberta, Canada; 5 Campus Saint-Jean, University of Alberta, Edmonton, Alberta, Canada; 6 Coppin Center for Organic Synthesis, Coppin State University, Baltimore, Maryland, United States of America; I2MC INSERM UMR U1048, France

## Abstract

**Background:**

Insulin resistance and early type-2 diabetes are highly prevalent. However, it is unknown whether Intralipid® and sevoflurane protect the early diabetic heart against ischemia-reperfusion injury.

**Methods:**

Early type-2 diabetic hearts from Sprague-Dawley rats fed for 6 weeks with fructose were exposed to 15 min of ischemia and 30 min of reperfusion. Intralipid® (1%) was administered at the onset of reperfusion. Peri-ischemic sevoflurane (2 vol.-%) served as alternative protection strategy. Recovery of left ventricular function was recorded and the activation of Akt and ERK 1/2 was monitored. Mitochondrial function was assessed by high-resolution respirometry and mitochondrial ROS production was measured by Amplex Red and aconitase activity assays. Acylcarnitine tissue content was measured and concentration-response curves of complex IV inhibition by palmitoylcarnitine were obtained.

**Results:**

Intralipid® did not exert protection in early diabetic hearts, while sevoflurane improved functional recovery. Sevoflurane protection was abolished by concomitant administration of the ROS scavenger N-2-mercaptopropionyl glycine. Sevoflurane, but not Intralipid® produced protective ROS during reperfusion, which activated Akt. Intralipid® failed to inhibit respiratory complex IV, while sevoflurane inhibited complex I. Early diabetic hearts exhibited reduced carnitine-palmitoyl-transferase-1 activity, but palmitoylcarnitine could not rescue protection and enhance postischemic functional recovery. Cardiac mitochondria from early diabetic rats exhibited an increased content of subunit IV-2 of respiratory complex IV and of uncoupling protein-3.

**Conclusions:**

Early type-2 diabetic hearts lose complex IV-mediated protection by Intralipid® potentially due to a switch in complex IV subunit expression and increased mitochondrial uncoupling, but are amenable to complex I-mediated sevoflurane protection.

## Introduction

Type-2 diabetes mellitus is the most prevalent form of diabetes (95%) and is characterized by elevated fasting blood glucose levels due to insulin resistance [1]. Diabetic patients undergoing cardiac and non-cardiac surgery suffer frequently from myocardial dysfunction and infarction, cerebral stroke, and renal dysfunction. The diabetic heart is already jeopardized by detrimental metabolic derangements and thus at high risk of low cardiac output and heart failure [2]. Mortality after myocardial infarction and cardiac surgery is double in diabetic patients [3], [4]. The cause for these complications in diabetic patients is the greater sensitivity of diabetic hearts to ischemia-reperfusion injury. This has been linked to impaired Akt signaling and GLUT4 trafficking, as well as mitochondrial dysfunction [5], [6], [7], [8].

Rahman and colleagues previously reported marked protection of the heart against ischemia-reperfusion injury with a 70% reduction in infarct size when Intralipid® was administered in high doses (1%) at the onset of reperfusion [9]. Administration of Intralipid® activates protection signaling pathways (ERK1/2, Akt and GSK3β) and inhibits the mitochondrial permeability transition pore [10]. Recently, our group confirmed these results and discovered that palmitoylcarnitine, the active fatty acid product of Intralipid®, increases reactive oxygen species (ROS) production at early reperfusion via the inhibition of complex IV of the respiratory chain, and activates reperfusion injury salvage kinases in healthy rat hearts [11]. However, whether Intralipid® can still trigger protection against ischemia-reperfusion injury in the context of insulin resistance and early type-2 diabetes is currently unknown. Impairment or complete loss of cardioprotection by pharmacological agents is well known in many diabetic models [12]. Here, we hypothesized that Intralipid® would not trigger ROS signaling at early reperfusion and hence elicit no protection in diabetic hearts. We compared the Intralipid® effects with those of sevoflurane, a potentially protective volatile anesthetic [13], [14]. For our experiments, we have chosen the fructose-fed rat model [15], which is a dietary model of the metabolic syndrome and of the early changes associated with type-2 diabetes.

## Materials and Methods

All materials were from Sigma-Aldrich Canada (Oakville ON, Canada) unless otherwise stated.

### Animal ethics statement

The investigation conforms to the Guide for the Care and Use of Laboratory Animals published by the US National Institutes of Health (NIH Publication, 8th Edition, 2011) and was approved by the University of Alberta Animal Policy and Welfare Committee.

### Dietary model of type-2 diabetes mellitus using fructose-feeding [16]

Male Sprague-Dawley rats (8 weeks old, from the Biosciences breeding colony, University of Alberta, Edmonton, Canada) were fed for 6 weeks with standard chow (49% maize farina, protein 23.4%, fat 10%; PicoLab® Laboratory Rodent diet 5LOD) combined with 10% fructose dissolved in the drinking water. The healthy control group consisted of age-matched rats fed with standard chow and tap water. After 6 weeks of fructose feeding, glucose, insulin and triglyceride blood concentrations were determined and compared to age-matched rats fed with standard chow. Briefly, after overnight fasting, 200 µL blood samples were collected into heparinized tubes (Natelson micro blood collecting tubes, Fisher Scientific, Ottawa ON, Canada) and plasma aliquots were stored at −20°C. Insulin in plasma was measured using the Insulin (rat) Ultrasensitive ELISA immunoassay (ALPCO Diagnostics, Salem NH, USA), triglycerides in plasma were measured by the WAKO Triglyceride E Kit method (Wako Chemicals, Richmond VA, USA), and glucose in plasma was measured using PGO enzymes and o-dianisidine as a colorimetric substrate (Sigma-Aldrich). The quantitative insulin-sensitivity check index (QUICKI) was calculated from fasting glucose (in mg/dL) and fasting insulin concentrations (in µIU/mL) as follows: QUICKI  =  1/(log [fasting insulin] + log [fasting glucose]) [17].

### Working heart perfusions

Rats (14 weeks of age) were anesthetized with pentobarbital (150 mg/kg, i.p.). Each heart was rapidly removed and perfused initially in a non-working Langendorff mode with Krebs-Henseleit solution for 10 min. The working mode perfusion was subsequently established (11.5 mmHg preload, 80 mmHg afterload, 5 Hz paced rate) with a recirculating perfusate of 100 mL (37°C, pH 7.4) gassed with 95% O_2_/5% CO_2_ mixture that consisted of a modified Krebs-Henseleit solution containing (mmol/L): KCl (4.7), NaCl (118), KH_2_PO_4_ (1.2), MgSO_4_ (1.2), CaCl_2_ (2.5), NaHCO_3_ (25), glucose (11), palmitate (1.2, pre-bound to 3% bovine serum albumin) and insulin 100 mU/L [18]. All hearts were subjected to 15 min of 37°C zero-flow ischemia and 30 min of reperfusion (IR). Cardiac output (mL/min) and aortic flow (mL/min) were measured using ultrasonic flow probes (Transonic T206, Transonic Systems Inc., Ithaca, NY) placed in the left atrial inflow and the aortic outflow lines. Left ventricular work (mL/min·mmHg) was calculated as LVW  =  cardiac output • (aortic systolic pressure − preload). Coronary flow (ml/min) was calculated as the difference between cardiac output and aortic flow. Measurements were averaged for the pre- and postischemic periods. Hearts were randomly assigned to the following groups: (1) untreated diabetic hearts (ff-IR) (2) diabetic hearts treated with Intralipid®1% (Baxter, Mississauga ON Canada) administered at the time of reperfusion, ff-IR/IL) [11] (3) diabetic hearts treated with sevoflurane (2 vol.-%, bubbled into the perfusate) administration 15 min before ischemia and during reperfusion (ff-IR/SEV) [19]. Additional two groups served to assess the involvement of ROS, namely diabetic hearts treated with sevoflurane (2 vol.-%) plus concomitant N-2-mercaptopropionyl glycine (MPG; 10 µM) (ff-IR/SEV+MPG) and diabetic hearts treated with MPG (10 µM) alone administered 15 min before ischemia and during reperfusion (ff-IR/MPG). To study signaling events, additional experiments were performed with groups of diabetic hearts reperfused for only 3 or 10 min. Finally, 1 µM palmitoylcarnitine (ff-IR/C16∶0c) was added to the perfusate at the time of reperfusion in separate experiments to attempt the rescue of Intralipid® protection in diabetic hearts [11]. Additional diabetic hearts were perfused aerobically to measure the effects of Intralipid®on mitochondrial respiratory chain complex activities and to quantify the acylcarnitine tissue content. Some diabetic hearts were used to collect apical fibers to measure inhibitor titration curves for complex IV (cytochrome c oxidase) and mitochondrial hydrogen peroxide (H_2_O_2_) release. These data were compared with results measured in healthy age-matched hearts [11]. All hearts were immediately frozen in liquid nitrogen with Wollenberger clamps and stored at −80°C for subsequent molecular analyses.

### Aconitase assay for the determination of ROS production in mitochondrial matrix of perfused hearts

Aconitase activity was evaluated according to the protocols of Gardner et al. [20] in mitochondria isolated from perfused hearts collected after 3 min of reperfusion. Mitochondria were isolated in the presence of 5 mM sodium citrate to protect aconitase from oxidation. Mitochondrial preparations were diluted to 0.1 mg/ml in 50 mM Tris-HCl, pH 7.4 containing 0.05% Triton X-100. Aconitase activity was assayed as the rate of NADP^+^ reduction (extinction coefficient for NADPH at 340 nm is 0.00622 µM^−1^ ⋅ cm^−1^) at 37°C in a reaction mixture containing 50 mM Tris-Cl (pH 7.4), 5 mM sodium citrate, 0.6 mM MnCl_2_, 0.2 mM NADP^+^, 1 U/ml isocitrate dehydrogenase, and 0.1 mg/ml mitochondrial protein. One unit of aconitase converts 1.0 nmol of citrate to isocitrate per minute at 37°C.

### Citrate synthase (CS) activity

As a measure of mitochondrial content, the activity of the mitochondrial matrix marker enzyme citrate synthase (CS) was measured at 412 nm by monitoring the formation of thionitrobenzoate, as previously described [21].

### Amplex Red assay for the determination of mitochondrial H_2_O_2_ release in permeabilized cardiac fibers

To assess mitochondrial H_2_O_2_ emission capacity from collected cardiac fibers, we determined H_2_O_2_ production under non-respiring conditions (no ADP) and under active oxidative phosphorylation (5 mM ADP) using a combination of pyruvate (5 mM)/malate (2 mM) and succinate (10 mM) as substrates to ensure full operation of the citric acid cycle, as well as to provide both NADH and FADH_2_ to feed electrons through complexes I and II, respectively, of the mitochondrial electron transport chain. H_2_O_2_ production was measured using the Amplex Red method (Invitrogen, Carlsbad, CA) [22]. Horseradish peroxidase (HRP; 2 U/ml) catalyzed the reaction between Amplex Red (20 µM) and H_2_O_2_ in the presence of exogenously added superoxide dismutase (10 U/ml), forming the fluorophore resorufin, which was monitored at excitation/emission wavelengths of 540/590 nm. H_2_O_2_ standard curves were generated for each independent experiment, to calculate the cumulative mitochondrial H_2_O_2_ production from the resorufin signal. H_2_O_2_ production at each time point was then determined by calculating the rate of change in H_2_O_2_ concentration over 20 min. Background rates of fluorescence change in the absence of added substrates were subtracted for each experiment. At the end of each experiment, cardiac fibers were homogenized for CS activity assays. H_2_O_2_ production rate was expressed as pmol/min/IU CS activity.

### High-resolution respirometry in permeabilized cardiac fibers

Respiration measurements were performed in saponin-permeabilized fibers prepared from freshly excised left ventricular apex of of either perfused or unperfused hearts, using the Oroboros Oxygraph 2K system (Oroboros, Innsbruck, Austria) [11], [18]. Measurements were conducted in an assay buffer comprising 110 mM sucrose, 60 mM K-lactobionate, 20 mM taurine, 0.5 mM EGTA, 3 mM MgCl_2_·6H_2_O, 10 mM KH_2_PO_4_, 20 mM HEPES, and 1 g/L bovine serum albumin (pH 7.1, 30°C). Mitochondrial respiration was measured in the presence of 5 mM ADP or absence of ADP (leak respiration) using substrates for complexes I, II, III, and IV as follows. Complex I: pyruvate (5 mM)/malate (2 mM); complex II: succinate (10 mM); complex III: decylubiquinol (0.5 mM); complex IV: ascorbate (2 mM)/tetramethylphenylenediamine dihydrochloride (TMPD; 0.5 mM). Substrates used together with the inhibition of complex I by rotenone (0.5 µM), complex II by malonate (2 mM) complex III by antimycin A (2.5 µM), and complex IV by azide (100 mM) provided complex-specific flux measurements. All respiratory data were normalized to CS activity as a marker for mitochondrial content (nmol O/s/CS). Inhibition of complex IV by palmitoylcarnitine was assessed by measuring oxygen consumption in the presence of ascorbate/TMPD and 5 mM ADP and titrating palmitoylcarnitine. To measure the effects of sevoflurane on mitochondrial respiratory chain complex activities, saponin-permeabilized fibers freshly prepared from early diabetic hearts were exposed to 0.35 mM sevoflurane dissolved in dimethyl sulfoxide, equivalent to the clinically used concentration of 1 MAC (minimum alveolar concentration) sevoflurane [23]. Rates of oxygen consumption were normalized to those measured in the presence of dimethyl sulfoxide alone (solvent control).

### Determination of tissue triglyceride content

After chloroform/methanol extraction of lipids from cardiac tissue, triglyceride content was quantified colorimetrically with the enzymatic assay kit L-Type Triglyceride M (Wako Pure Chemical Industries, Richmond VA, USA). The colorimetric assay was performed in the presence of 3∶2 tert-butyl alcohol:Triton X-100/methyl alcohol (1∶1) mixture.

### Mass spectrometry for acylcarnitine profiling

Tissue levels of acylcarnitine species were measured using electrospray ionization tandem mass spectrometry [24]. Acylcarnitines were extracted from heart tissue with methanol and quantified using eight isotopically labeled internal standards (Cambridge Isotopes Laboratories, Andover, MA). Precursor ions of m/z 85 in the mass range of m/z 150 to 450 were acquired on a PE SCIEX API 365 LC-ESI-MS/MS instrument (AppliedBiosystems, Foster City, CA) [24].

### Carnitine palmitoyl transferase (CPT) activity

Total CPT (CPT-I and CPT-II) activity was determined using mitochondria isolated from frozen heart tissues, according to Boudina et al. [25]. The reaction was performed with 20 µg mitochondria in a total of 200 µL reaction volume at 37°C. CPT-II activity was measured using an identical reaction as for total CPT activity, but in the presence of 10 µM malonyl-CoA to inhibit CPT-I activity. CPT-I activity was then calculated as the difference between total CPT and CPT-II activity.

### Immunoblotting

Frozen heart tissue was homogenized in ice-cold buffer containing 50 mM Tris (pH 8.0), 150 mM NaCl, and 1% NP-40, and supplemented with protease and phosphatase inhibitor cocktail mix (Sigma-Aldrich). The homogenate was centrifuged at 1000 g for 10 min at 4°C. The resulting supernatant was collected for protein concentration determination and used for immunoblotting in sodium dodecyl sulfate polyacrylamide gel electrophoresis. Protein concentration was measured by Bradford assay. The primary antibodies (Akt, pAkt, ERK1/2, pERK1/2, all from Cell Signaling Technology, Danvers, MA) were polyclonal rabbit antibodies. Mouse monoclonal anti-COX IV antibody (clone 20E8C12, ab14744, mitochondrial loading control), polyclonal rabbit anti-COXIV isoform 2 (ab70112), and polyclonal rabbit anti-UCP3 (ab3477) were purchased from Abcam Inc. (Cambridge, MA, USA). Polyclonal goat anti-ANT (N-19) was purchased from Santa Cruz Biotechnology Inc. (Dallas, TX, USA). Immunoreactivity was visualized by horseradish peroxidase-conjugated antibodies using a peroxidase-based chemiluminescence detection kit (ECL) (PerkinElmer, Woodbridge, Ontario, Canada). The intensity of the bands was quantified by two individuals (PHL, EL) independent of each other using ImageJ software (http://rsbweb.nih.gov/ij/).

### Statistical analysis

Values are given as mean (SD) [or mean (SEM) for the concentration-response curves of complex IV inhibition to avoid overlap] or median (25^th^, 75^th^ percentile) depending on the underlying data distribution for the indicated number of independent observations. The significance of differences in hemodynamic variables among groups was determined by repeated measures analysis-of-variance (RM-ANOVA) followed by the Student-Newman-Keuls method for posthoc analysis. For comparisons involving two independent groups, Student t-test or Mann-Whitney rank sum test (two-tailed) were used, depending on the underlying data distribution. For comparisons involving multiple groups, analysis-of-variance (ANOVA) followed by the Student-Newman-Keuls Multiple Comparison Procedure was used. Differences are considered significant if p<0.05. SigmaPlot (version 12.0; Systat Software, Inc., Chicago, IL) was used for the analyses. The inhibitory effect of palmitoylcarnitine on respiratory chain complex IV activity was measured by the global curve fitting routine implemented in SigmaPlot. Comparison between concentration-response curves and concentration-response parallelism (i.e., one curve shifted to the right or up as compared to the other) were measured using the F-test [26].

## Results

After 6 weeks of fructose feeding, rats reliably exhibited increased fasting plasma glucose, insulin, and triglyceride levels and reduced quantitative insulin sensitivity check index values consistent with type-2 diabetes (Table 1) [17]. In addition, fructose-fed rats showed increased diastolic and systolic blood pressure (Table 1). Hearts of fructose-fed rats exhibited fat overload as measured by triglyceride tissue content and C16∶1/C16 and C18∶1/C18 ratios (“desaturation index”) (Table 1) [27].

**Table 1:** Effects of fructose feeding on physiological and metabolic parameters.

	healthy	ff	P-value
Initial body weight [g]	250 (7)	251 (8)	0.76
Final body weight [g]	558 (29)	520 (36)	**0.015**
Heart wet weight/body weight	0.0040 (0.0038; 0.0042)	0.0040 (0.0038; 0.0040)	0.303#
Systolic blood pressure [mmHg]	117 (6)	148 (7)	**<0.001**
Diastolic blood pressure [mmHg]	68 (7)	103 (11)	**<0.001**
Plasma fasting glucose [mg*dL^−1^]	114 (110; 116)	143 (136; 150)	**<0.001**
Plasma fasting insulin [ng*mL^−1^]	0.42 (0.18)	1.12 (0.34)	**<0.001**
QUICKI	0.32 (0.02)	0.27 (0.01)	**<0.001**
Plasma triglycerides [mg*mL^−1^]	74 (71; 102)	161 (147; 187)	**<0.001**
Cardiac tissue triglycerides [µmol/g dry wt]	6.35 (2.01)	7.74 (2.29)	**0.016**
C16∶1c/C16∶0c desaturation ratio	0.34 (0.04)	0.57 (0.20)	**0.024**
C18∶1c/C18∶0c desaturation ratio	7.81 (0.82)	9.64 (0.93)	**0.011**

Data are presented as mean (SD) or median (25^th^; 75^th^ percentile). N = 8–16 (N = 30 for tissue triglycerides). # Mann-Whitney Rank Sum Test.

healthy, age-matched control rats (fed standard chow and water).

ff, rats fed standard chow and 10% fructose added to the drinking water.

QUICKI, quantitative insulin sensitivity check index.

C16∶1c, cardiac tissue levels of palmitoleoylcarnitine.

C16∶0c, cardiac tissue levels of palmitoylcarnitine.

C18∶1c, cardiac tissue levels of oleoylcarnitine.

C18∶0c, cardiac tissue levels of stearoylcarnitine.

### Early type-2 diabetic hearts lose protection by Intralipid® but not by sevoflurane against ischemia-reperfusion injury

**Figure 1:**
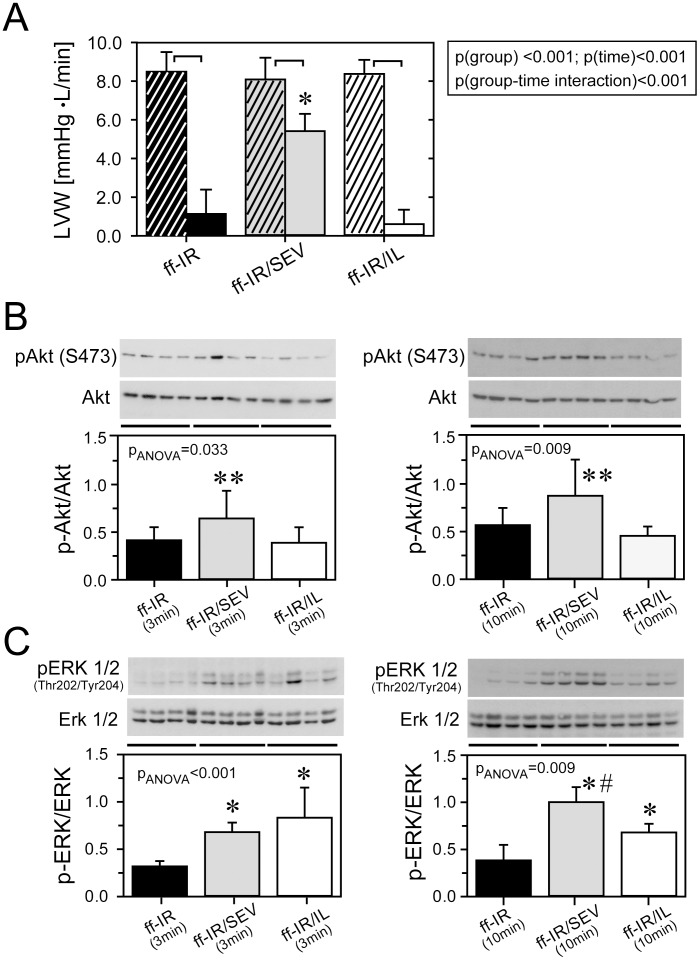
Left ventricular work and protection signaling in Intralipid®- and sevoflurane-treated early type-2 diabetic hearts. Panel A: average left ventricular work (LVW) during equilibration (striped columns) and reperfusion (30 min: solid columns) in untreated early diabetic hearts (ff-IR: N = 10), early diabetic hearts exposed to 2 vol.-% sevoflurane (ff-IR/SEV; N = 10), and early diabetic hearts treated with 1% Intralipid® at the onset of reperfusion (ff-IR/IL; N = 6). Data are mean ± SD. Panel B: p-Akt (at Ser473) to total Akt immunoblots from tissue samples collected 3 min and 10 min after the onset of reperfusion. Panel C: p-ERK1/2 (at Thr202/Tyr204) to total ERK1/2 immunoblots from the same tissues. ff-IR(time), untreated hearts exposed to 15 min of ischemia and 3 min (ff-IR/3 min) or 10 min (ff-IR/10 min) of reperfusion, respectively; ff-IR/SEV(time), hearts exposed to ff-IR(time) and 2 vol.-% sevoflurane; ff-IR/IL(time), hearts exposed to ff-IR(time) and 1% Intralipid® at the onset of reperfusion. *, significantly different from ff-IR(time); **, significantly different from ff-IR(time) and ff-IR/IL(time); #, significantly different from ff-IR/IL(time). Data are mean ± SD. N = 4 hearts in each group.

We evaluated the impact of Intralipid® administration on recovery of LVW in early diabetic hearts, and compared this effect with that of the volatile anesthetic sevoflurane, which has been shown to be effective in healthy and diseased hearts [13], [18]. Early diabetic hearts were subjected to 15 min of ischemia and 30 min of reperfusion. Administration of Intralipid® at the onset of reperfusion did not provide any protection (Figure 1A and Table S1). Intralipid®-treated early diabetic hearts also showed significant reduction in postischemic coronary perfusion (Table S1). In contrast, peri-ischemic administration of 2 vol.-% sevoflurane to the perfusate 15 min before ischemia and during reperfusion markedly improved functional recovery, showing its preserved ability to protect early diabetic hearts against ischemia-reperfusion injury (Table S1). Contrary to the effects of Intralipid® treatment, coronary perfusion was preserved in sevoflurane-treated hearts (Table S1). Sevoflurane-protected early diabetic hearts showed increased oxidative phosphorylation compared to unprotected, i.e. Intralipid®-treated, hearts as measured by mitochondrial oxygen consumption using both glucose- and fatty acid-derived substrates (Table 2). Similar to our previous findings in healthy hearts [11], Intralipid® did not contribute to substrate metabolism in the early diabetic heart (Figure S1).

**Table 2:** Mitochondrial respiration in saponin-skinned cardiac fibers harvested at the end of the ischemia-reperfusion protocols.

Protocol	Substrates	ff-IR	ff-IR/IL	ff-IR/SEV	P-value
Glucose oxidation protocol	pyruvate/malate	11.4 (1.9)	11.5 (2.0)	15.8 (2.2)*	**<0.001**
Fatty acid oxidation protocol	palmitoylcarnitine/malate	3.0 (1.0)	2.6 (0.5)	3.6 (0.8)#	**0.028**

Mitochondrial respiration was measured in the presence of glucose-derived (pyruvate/malate) or fat-derived substrates (palmitoylcarnitine/malate). The measured oxygen consumption (normalized to citrate synthase activity) is expressed as nmol O_2_ s^−1^/CS. Data are presented as mean (SD). *, significantly increased compared to all other groups;#, significantly increased compared to ff-IR/IL.

ff-IR, hearts from fructose-fed rats exposed to ischemia-reperfusion (IR) without treatment (N = 10); ff-IR/SEV, hearts from fructose-fed rats exposed to IR with sevoflurane (2 vol.-%) conditioning (N = 10); ff-IR/IL, hearts from fructose-fed rats exposed to IR with Intralipid (1%) treatment at the onset of reperfusion (N = 6).

### Differential ROS-dependent activation of reperfusion injury salvage kinases (RISK) in Intralipid®- and sevoflurane-treated early type-2 diabetic hearts

**Figure 2:**
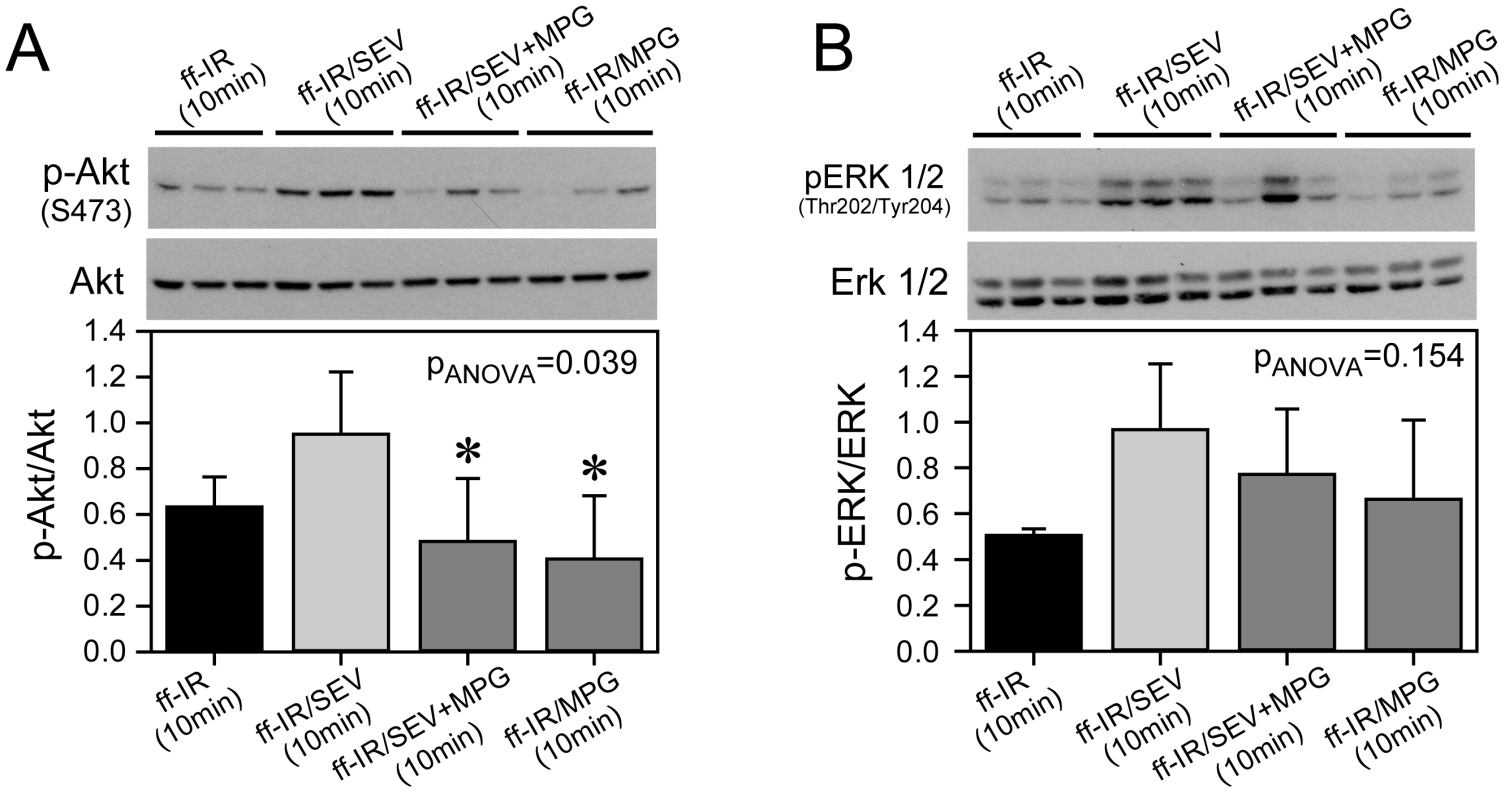
ROS-dependent protection signalling in sevoflurane-treated early type-2 diabetic hearts. Panel A: representative immunoblots showing blunted activation of Akt in early diabetic hearts subjected to 15 min of ischemia and 10 min reperfusion in the presence of 2 vol.-% sevoflurane and concomitantly treated with the antioxidant MPG. *, significantly different from sevoflurane-treated hearts. Panel B: ERK activation is not mediated by ROS (same tissue samples as in Panel A). ff-IR(10 min), untreated hearts exposed to 15 min of ischemia and 10 min of reperfusion; ff-IR/SEV(10 min), hearts exposed to ff-IR(10 min) and 2 vol.-% sevoflurane; ff-IR/SEV+MPG(10 min), sevoflurane-treated hearts exposed to 15 min of ischemia and 10 min of reperfusion with N-(2-mercaptopropionyl) glycine (MPG; 10((M). ff-IR/MPG(10 min), hearts exposed to 15(min of ischemia and 10(min of reperfusion in the presence of MPG alone. Data are mean ± SD. N = 4–6 hearts in each group.

Next, we wanted to know whether protection by sevoflurane would be ROS-dependent and whether reperfusion injury salvage kinases would be differentially activated in Intralipid®- and sevoflurane-treated early diabetic hearts. Functional recovery by sevoflurane was abolished by concomitant administration of MPG, a reactive oxygen species scavenger (LVW(equilibration) =  8.1±1.5 mmHg*L/min; LVW(reperfusion) =  1.8±0.6 mmHg*L/min; please see Table S1). The temporal activation of Akt and ERK1/2 in tissue samples collected at 3 and 10 min of reperfusion was measured by immunoblotting. Akt phosphorylation was significantly increased in 3 and 10 min samples of sevoflurane-treated but not Intralipid®-treated diabetic hearts (Figure 1B). ERK1/2 activity was increased in 3 and 10 min samples of both Intralipid®- and sevoflurane-treated hearts (Figure 1C). However, since MPG abolished activation of Akt but not ERK1/2 in sevoflurane-treated hearts, ERK1/2 appears not to be causally involved in cardioprotection of this model (Figure 2A and 2B). MPG alone did not affect Akt or ERK1/2.

### Loss of electron transport chain inhibition and ROS generation in Intralipid®- but not sevoflurane-treated early type-2 diabetic hearts

**Figure 3:**
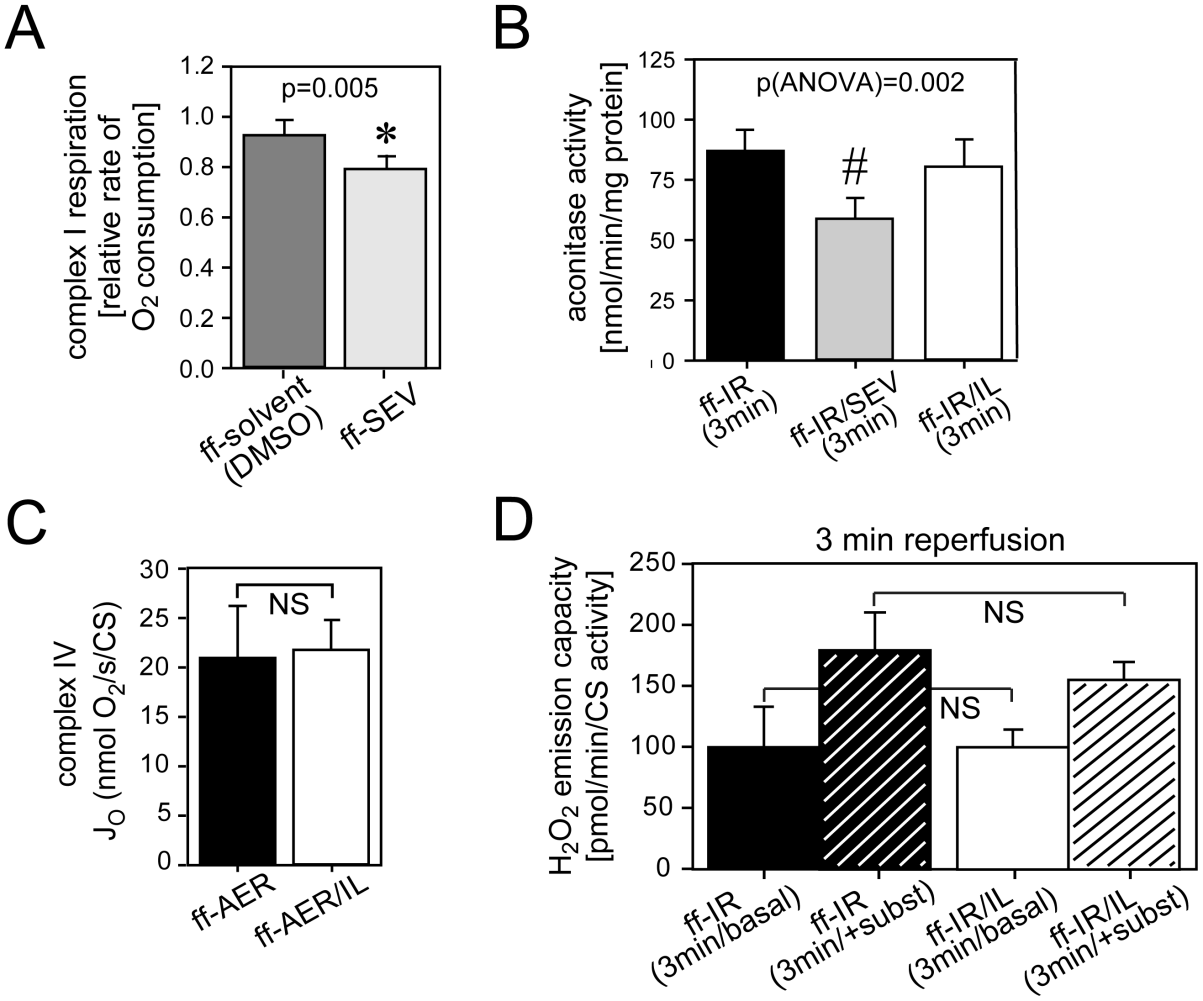
Respiratory chain inhibition and ROS production at the onset of reperfusion. Panel A: sevoflurane inhibits complex I in diabetic cardiac fibers. Polarographic measurements of oxygen consumption in sevoflurane-treated (0.35(mM) diabetic cardiac fibers oxidizing the complex I substrates pyruvate+malate. *, significantly different from solvent control. DMSO, dimethyl sulfoxide (0.1%) used as solvent for sevoflurane. Panel B: loss of aconitase activity in sevoflurane-treated but not in Intralipid®-treated early diabetic hearts. #, significantly different from untreated and Intralipid®-treated early diabetic hearts. Panel C: polarographic measurements of oxygen consumption in cardiac fibers collected from Intralipid®-treated diabetic hearts oxidizing the complex IV substrates N,N,N′,N′-tetramethyl-p-phenylenediamine/ascorbate. ff-AER, hearts with time-matched aerobic perfusion. ff-AER/IL, hearts with time-matched aerobic perfusion treated with 1% Intralipid®. Panel D: hydrogen peroxide (H_2_O_2_) emission capacity during early reperfusion. ff-IR(3 min), untreated hearts exposed to 15 min of ischemia and 3 min of reperfusion; ff-IR/IL(3 min), hearts exposed to 15 min of ischemia and 3 min of reperfusion treated with 1% Intralipid® at the onset of reperfusion. Basal, without substrates. +subst, with added substrates (pyruvate/malate/succinate). Data are mean ± SD. N = 4–5 hearts per group.

Previous work has shown that the formation of ROS during early reperfusion is the unifying mediator of cardioprotection by Intralipid® [11] and sevoflurane [19], [28] in healthy hearts. In the case of sevoflurane, ROS is produced at complex I of the respiratory chain via the attenuation of complex I activity [29], [30], [31], a result that was confirmed and extended in this study to early diabetic hearts (Figure 3A). Sevoflurane attenuated complex I activity of early diabetic hearts (for complete data on mitochondrial respiration in the presence of sevoflurane, see Table S2) and significantly reduced aconitase activity in tissue samples collected after 3 min of reperfusion, consistent with increased formation of ROS (Figure 3B). As for Intralipid®, we previously demonstrated that ROS formation in healthy hearts is the result of complex IV inhibition [11]. When we assessed the respiratory complex activities in time-matched aerobically perfused early diabetic hearts, we did not observe any inhibition of complex IV activity by Intralipid® (Figure 3C) (for complete respiratory complex activities of time-matched aerobically perfused diabetic hearts see Table S3). In healthy hearts, Intralipid®significantly inhibited complex IV by 27% [11]. There was no loss of aconitase activity in Intralipid®-treated diabetic hearts (Figure 3B) at early reperfusion, an observation which was further confirmed by Amplex Red assay (Figure 3D).

### Switch in subunit IV of cytochrome c oxidase and increased uncoupling as potential mechanisms underlying loss of Intralipid®-mediated cardioprotection in early type-2 diabetic hearts

**Figure 4:**
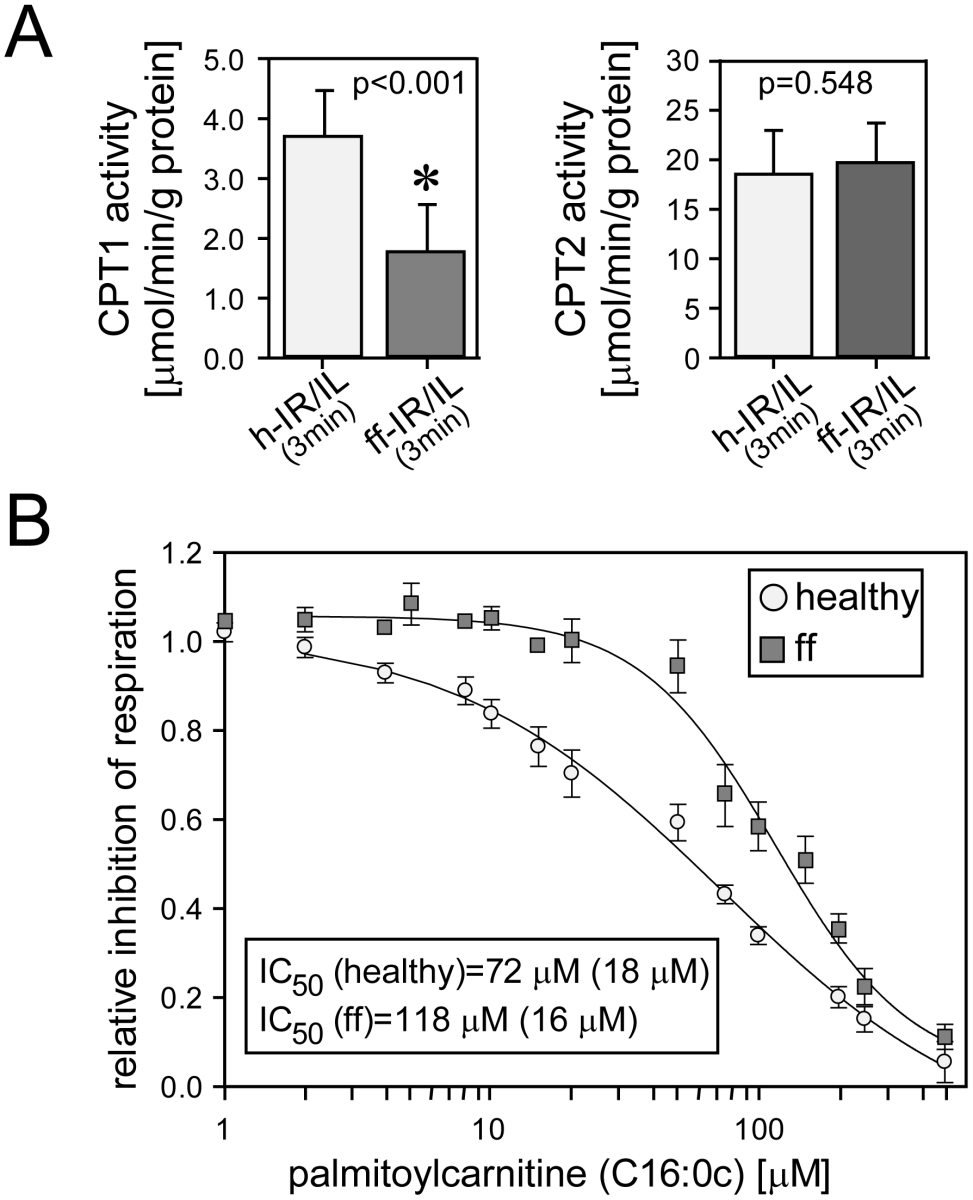
Mitochondrial fatty acid uptake and complex IV inhibition by palmitoylcarnitine in early type-2 diabetic hearts. Panel A: carnitine palmitoyltransferase 1 and 2 (CPT1 and CPT2, respectively) activity at the onset of reperfusion in healthy (h-IR/IL(3 min)) and early diabetic (ff-IR/IL(3 min) hearts treated with 1% Intralipid®. *, significantly different from healthy hearts. N = 10 hearts in each group. Panel B: concentration-dependent inhibition of complex IV by palmitoylcarnitine (C16∶0c) in permeabilized cardiac fibers of healthy (reproduced from reference [19]) and fructose-fed (ff) rats. Complex IV inhibition is given as relative decrease in oxygen consumption. IC_50_, concentration of palmitoylcarnitine that reduces the respiration rate by 50%. N = 5–6 hearts per group. Data are or mean ± SD (panels A) or mean ± SEM (panel B).

We previously identified the fatty acid intermediate palmitoylcarnitine as pivotal in Intralipid®-mediated cardioprotection in healthy hearts acting through complex IV inhibition and ROS generation [11]. We hypothesized that fatty acid uptake into mitochondria would be reduced in early diabetic hearts. Indeed, the enzymatic activity of carnitine palmitoyltransferase 1 was significantly reduced in early diabetic hearts reperfused in the presence of Intralipid® (Figure 4A). Reperfused early diabetic hearts also exhibited reduced mitochondrial uptake of the most abundant C18∶2 constituent of Intralipid®, when compared to healthy hearts (Figures S2 and S3, which provide an overview of the main finding from tissue acylcarnitine profiling). Aerobically perfused Intralipid®-treated diabetic hearts further exhibited reduced long-chain acylcarnitine tissue concentrations compared with healthy hearts, confirming reduced mitochondrial fatty acid uptake (please see Figure S3). To circumvent the fatty acid uptake limitation in fructose-fed rats, we supplied palmitoylcarnitine – which readily crosses the mitochondrial membrane – at the onset of reperfusion. Contrary to findings in healthy hearts, no enhancement of functional recovery was achieved with 1 µM palmitoylcarnitine (LVW(equilibration) = 8.5±0.3 mmHg*L/min; LVW(reperfusion) = 1.7±0.7 mmHg*L/min; n = 4). We then tested if palmitoylcarnitine could still inhibit complex IV in the same way as it does in healthy hearts [11]. Palmitoylcarnitine titrations revealed a significantly different concentration-inhibition curve (overall comparison p(F-test) = 0; left-right shift p(F-test) = 10^−7^; up-down shift p(F-test) = 1.6*10^−9^) in diabetic cardiac fibers, resulting in higher IC_50_ values in diabetic hearts compared to healthy hearts (Figure 4B) [11]. Interestingly, there was no difference in complex IV inhibition by cyanide (which binds to the heme a3-CuB binuclear center) between healthy and diabetic hearts (data presented in Figure S4), implying a different binding site for palmitoylcarnitine.

**Figure 5:**
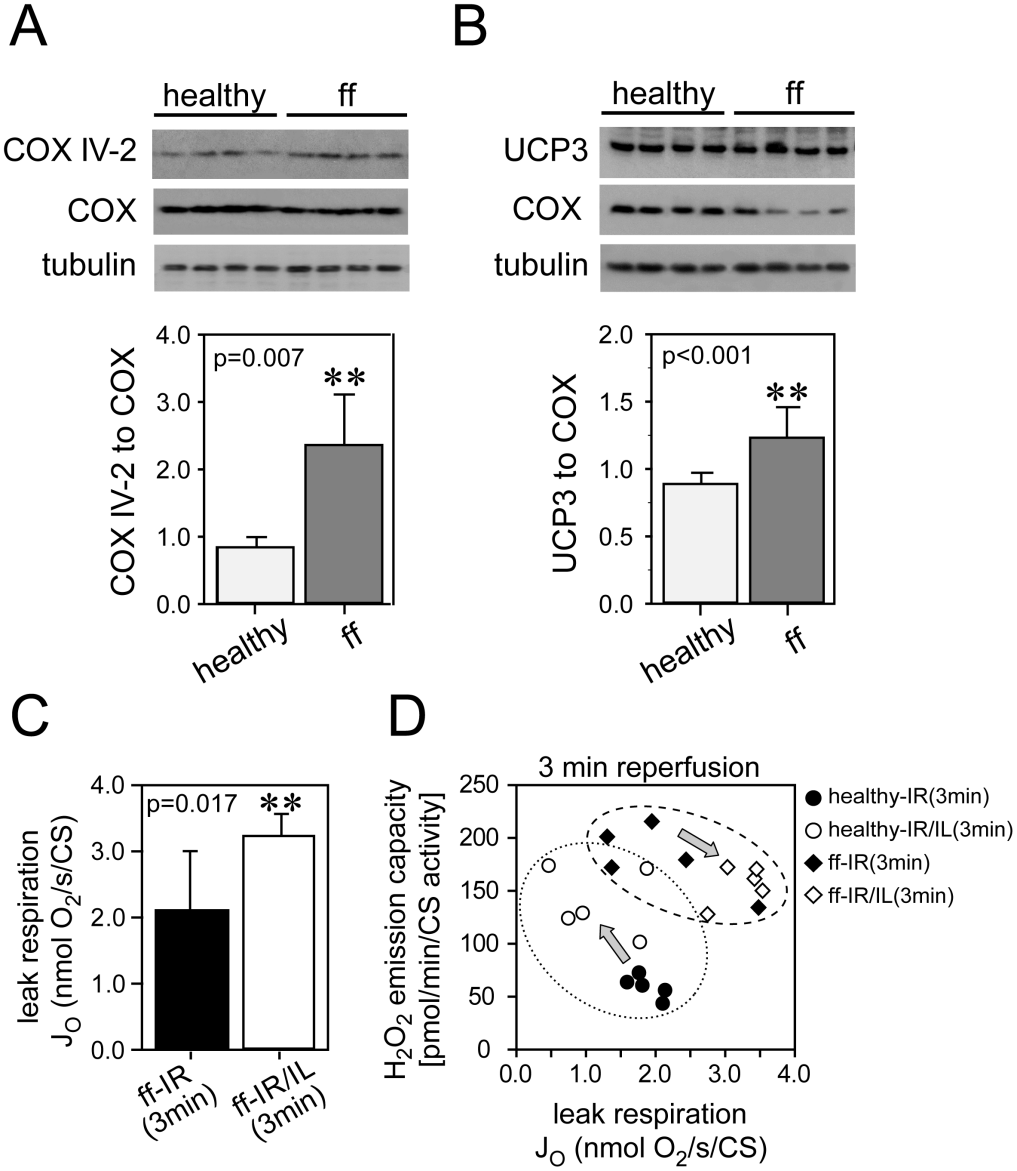
Mechanisms underlying loss of protective ROS signaling in early type-2 diabetic hearts. Panel A: increase in isoform 2 of complex IV (COX) subunit IV (COX IV-2) in cardiac mitochondria from fructose-fed (ff) rats as compared to healthy rats. Panel B: uncoupling protein 3 (UCP 3) is increased in cardiac mitochondria of fructose-fed (ff) rats as compared with healthy rats. COX, cytochrome c oxidase. Panel C: leak respiration (normalized to citrate synthase (CS) activity) with pyruvate as measured by polarography in permeabilized cardiac fibers from fructose-fed rats collected at reperfusion. Panel D: relationship between hydrogen peroxide (H_2_O_2_) emission capacity determined by Amplex Red assay and leak respiration (normalized to citrate synthase activity) with pyruvate measured by polarography in permeabilized cardiac fibers from healthy (reproduced from reference [19]) and fructose-fed (ff) rats collected at reperfusion. ff-IR(3 min), untreated hearts exposed to 15 min of ischemia and 3 min of reperfusion; ff-IR/IL(3 min), hearts exposed to 15 min of ischemia and 3 min of reperfusion with 1% Intralipid® at the onset of reperfusion; Arrows illustrate the opposing response to Intralipid® treatment in healthy vs. early diabetic hearts (see manuscript for details). **, significantly increased from healthy. Data are mean ± SD. N = 4–5 hearts in each group.

Early diabetic hearts expressed higher levels of subunit IV-2 of complex IV (Figure 5A), which was previously shown to enhance complex IV activity during metabolic stress [32], potentially accounting for the different inhibition characteristics. Uncoupling protein-3 levels were significantly increased in early diabetic hearts when normalized to nuclear encoded complex IV (Figure 5B). Increased levels of subunit IV-2 of complex IV and uncoupling protein-3 in diabetic hearts were also confirmed when normalized to adenine nucleotide translocase, a protein of the inner mitochondrial membrane (see Figure S5). Citrate synthase activity measurements in healthy control hearts and hearts of fructose-fed rats (17.2±2.2 vs 14.4±1.5 µmol/mL/min/µg, p<0.001, N = 20) confirmed the presence of fewer mitochondria in diabetic hearts. Evidence for enhanced uncoupling activity in fructose-fed rats can be seen in our experiments from (i) increased mitochondrial leak respiration in cardiac fibers of Intralipid®-treated early diabetic hearts collected at 3 min of reperfusion (Figure 5C); and (ii) increased leak respiration without changes in ROS production (Figure 5D). In contrast, Intralipid® treatment enhanced ROS production without increasing mitochondrial leak in healthy hearts (Figure 5D).

## Discussion

We chose the fructose-induced type-2 diabetes rat model because it reflects an early stage of diabetes due to its reversibility up to twelve weeks of feeding and the absence of severe maladaptive changes as observed in genetic, inbred or type-1 diabetes models [15]. Rats exposed to fructose-feeding for 6 weeks consistently exhibited characteristics of type-2 diabetes such as increased fasting glucose, hyperinsulinemia, hyperlipidemia, insulin resistance, and arterial hypertension [15]. In this dietary model of early type-2 diabetes, our study shows that Intralipid® treatment, a promising therapy against ischemia-reperfusion injury in healthy rats [11], completely lost its protection. We attribute this to the loss of Intralipid®-induced protective ROS signaling as a consequence of reduced sensitivity of complex IV to inhibition by palmitoylcarnitine and enhanced mitochondrial uncoupling. In contrast, sevoflurane, a clinically used drug, is still able to induce sufficient amounts of protective ROS via complex I inhibition in early diabetic hearts to activate reperfusion injury salvage kinases. Hence, ROS is a prerequisite to effective cardioprotection even in early diabetes, and its production depends on the impact of the cardioprotective agent on mitochondrial ROS production.

Metabolic inhibition at the level of the electron transport chain recently emerged as a unifying mechanism of cardioprotection [33]. At the onset of reperfusion, a surge of substrates and oxygen rapidly reestablish respiration causing a burst of ROS, Ca^2+^ overload and permeability transition pore opening. However, ROS can have both protective and deleterious effects depending on the time, location, and amount released. The burst of ROS released during reperfusion is associated with injury causing irreversible damage to proteins. Small amounts of ROS produced right at the onset of reperfusion have a signaling role and trigger cardioprotection against ischemia-reperfusion injury [34]. In the concept of “metabolic shut-down and gradual wake-up”, inhibitors of the electron transport chain slow down electron flux at early stages of reperfusion and thus facilitate an initial early peak of protective ROS well before the noxious ROS burst, which activates reperfusion injury salvage kinases and GSK3β, ultimately preventing mitochondrial permeability transition [19], [35]. While ischemic bouts inhibit complex I and II [33], volatile anesthetics specifically inhibit complex I [29], [30], [31]. We have recently shown that high-dose Intralipid® treatment through its intermediate palmitoylcarnitine specifically inhibits complex IV (as with nitric oxide, carbon monoxide, and hydrogen disulfide) in healthy hearts and provides protection by generation of protective ROS [11].

### How does palmitoylcarnitine inhibit complex IV and enhance ROS production in healthy hearts?

Our current experiments suggest − because of no differences in cyanide inhibition between diabetic and healthy hearts − that palmitoylcarnitine is inhibiting complex IV in healthy hearts via a mechanism distinct from that of cyanide's inhibition of complex IV. Fatty acids are known to bind to complex IV to directly modify its catalytic activity [36] or to modify the binding of ligands, such as cytochrome c [37], and to modulate electron flux in the electron transport chain. In fact, fatty acids, and many amphiphatic molecules which are sterically similar to acylcarnitines, bind to a conserved amphiphatic ligand binding region [38]. Complex IV inhibition per se does not increase ROS production from this complex itself, but more so from the reduction of redox centers in complex I or III [32]. It is thus possible that the amphiphatic palmitoylcarnitine binds to this recently identified regulatory site of complex IV. Alternatively, fatty acids (and possibly also their derivatives) modify cytochrome c binding to complex IV [37]. Oxidized cytochrome c is a powerful superoxide scavenger within the mitochondrial intermembrane space, and a shift from oxidized to reduced cyctochrome c, as expected by altered binding of cytochrome c to complex IV, would reduce ROS scavenging and increase ROS production.

### How does fructose-induced early type-2 diabetes abolish protective ROS signaling?

Mitochondria from early diabetic hearts show increased H_2_O_2_ levels compared to healthy mitochondria as measured by the Amplex Red assay [11], consistent with the concept of increased oxidative stress being a hallmark of insulin resistance and diabetes. Thus, the increased uncoupling protein-3 levels in diabetic mitochondria contribute to the maintenance of a normal membrane potential below the threshold of excessive ROS generation [39], [40]. Uncoupling proteins have been shown to be activated by ROS [41], products of lipid peroxidation [42], or fatty acids, albeit in vitro [43], [44] as oxidative stress-mitigating mechanism. Our data show increased leak respiration without changes in ROS production during early reperfusion in the presence of Intralipid®, and support the notion that fatty acids released from Intralipid® may greatly enhance the activity of uncoupling protein-3 (either directly or via lipid peroxidation), and thus efficiently annihilate the early increase in ROS production in diabetic mitochondria. Long-term exposure of myocytes to high glucose can impair complex IV activity via enzymatic O-GlcNAcylation [45]. Thus the diabetic heart may counteract this loss of complex IV activity by switching complex IV from subunit IV-1→IV-2, to directly increase the catalytic activity of complex IV. Increased levels of subunit IV-2 are associated with increased complex IV activity and reduced ROS production [46], [47]. Under normal conditions, there is very little subunit IV-2 relative to subunit IV-1 in the heart. However, under metabolic stress, i.e. increased ROS production or hypoxia [46], [48], [49], [50], the relative expression of IV-2/IV-1 is augmented as IV-2 expression is increased along with the rapid degradation of subunit IV-1. These findings potentially explain the observed difference in the inhibition characteristics by palmitoylcarnitine in our experiments.

### ROS signaling in the context of anesthetic-induced protection of diabetic hearts

We and others have shown that cardioprotection by volatile anesthetics is ROS-dependent [19], [51], [52], and we recently extended this paradigm to Intralipid®-treated cardioprotection [11]. Our current data on Intralipid®- and sevoflurane-mediated protection in early diabetic hearts confirm the importance of ROS in cardioprotection. Therefore, short-term administration of antioxidants during early stages of type-2 diabetes diminishes rather than restores cardioprotection. Although our results are different from an earlier study which reported loss of protection by sevoflurane postconditioning in the prediabetic state of the leptin-mutant Zucker obese rat model [53], the fact that protection in this model could not be rescued by cyclosporine A, a mPTP opener, points to severe downstream defects associated with this genetic mutant, which may not necessarily reflect conditions in early diabetes.

Pre- and postischemic administration of sevoflurane mimics the clinical situation where volatile anesthetics are usually given before and after a potentially ischemic challenge to the heart [54]. Although Intralipid® is unable to protect the diabetic heart against ischemia-reperfusion injury, sevoflurane may be still protective in patients with early type-2 diabetes. This may be particularly true in patients with reasonably well-controlled metabolism, which is indeed the case for the majority of surgical patients.

### Limitations of the study

It is possible that the timing of sevoflurane administration might have also contributed to the more efficient protection of sevoflurane compared with Intralipid®. However, since pre- and postischemic sevoflurane administration in diabetic hearts did not produce higher ROS levels than Intralipid® 3 min after the onset of reperfusion in healthy myocardium, as evidenced by a 25% loss of aconitase activity [11], it appears unlikely that the preischemic component of sevoflurane administration, i.e. the timing itself, accounts for sevoflurane's more efficient protection in diabetic hearts. Also, pre-ischemic Akt activity in sevoflurane-treated diabetic hearts showed no difference compared to untreated hearts, emphasizing the importance of Akt activation during early reperfusion. In fact, previous reports in healthy hearts show equal protection by postischemic vs pre- and postischemic sevoflurane administration in rat hearts in vivo [55]. Whether this also applies to early diabetic hearts needs further investigation.

### In summary

Our experiments show that effective cardioprotection by Intralipid® is lost in early type-2 diabetes, whereas sevoflurane still retains its beneficial properties. We discover that effective cardioprotection in early type-2 diabetes depends on the inhibition site of the electron transport chain and the impact of the cardioprotective agent on mitochondrial ROS production.

## Supporting Information

Figure S1 Glucose and fatty acid oxidation in rat hearts perfused with/without Intralipid.
(PDF)

Figure S2 Linoleoylcarnitine (C18∶2), oleoylcarnitine (C18;1), palmitoylcarnitine (C16∶0) levels as well as ratio between total tissue acylcarnitines (AC) and free carnitine in hearts from healthy (reproduced from reference 19 with permission) and fructose-fed rats subjected to 15 min ischemia and 3 min of reperfusion with/without 1% Intralipid® at the onset of reperfusion.
(PDF)

Figure S3 Linoleoylcarnitine (C18∶2), oleoylcarnitine (C18;1), palmitoylcarnitine (C16∶0) levels as well as ratio between total tissue acylcarnitines (AC) and free carnitine in hearts from healthy and fructose-fed rats aerobically perfused with/without 1% Intralipid®.
(PDF)

Figure S4 KCN titration experiments of cytochrome c oxidase activity.
(PDF)

Figure S5 Isoform 2 of complex IV and uncoupling protein 3 protein levels in healthy and diabetic hearts normalized to adenine nucleotide translocase.
(PDF)

Table S1 Hemodynamic data (full protocols; 30 min reperfusion time).
(PDF)

Table S2 Assessment of mitochondrial respiratory chain function in cardiac fibers from diabetic animals exposed to 2 vol.-% sevoflurane in the oxygraph chamber.
(PDF)

Table S3 Assessment of mitochondrial respiratory chain function in hearts from diabetic rats aerobically perfused with 1% Intralipid®.
(PDF)
