# Supplementary material for: Loss of Intralipid®- but Not Sevoflurane-Mediated Cardioprotection in Early Type-2 Diabetic Hearts of Fructose-Fed Rats: Importance of ROS Signaling
Source: PLoS One. 2014 Aug 15;9(8):e104971. doi: 10.1371/journal.pone.0104971 (PMC4134246; doi:10.1371/journal.pone.0104971)
Supplement: Figure S1 — Glucose and fatty acid oxidation in rat hearts perfused with/without Intralipid. (PDF) [file pone.0104971.s001.pdf]

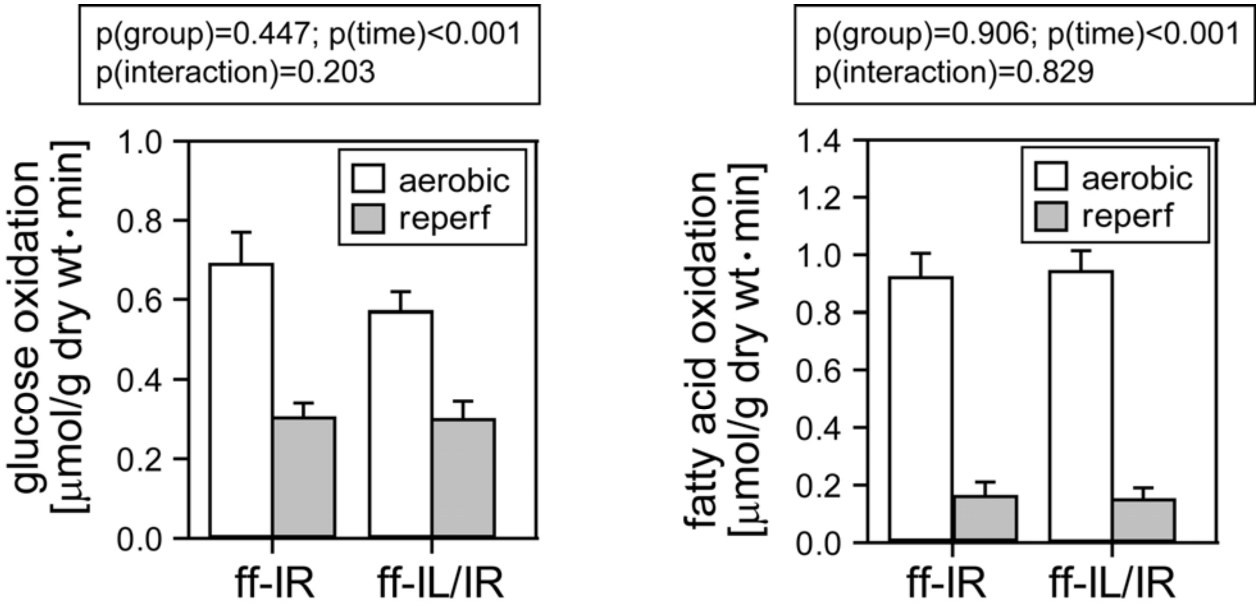

Figure S1: Glucose and fatty acid oxidation were determined by perfusing hearts with [U-^{14}C]glucose and [9,10-^{3}H]palmitate, respectively. Total myocardial $^{14}CO_2$ production and $^{3}H_2O$ production were determined every 10 min. Rates expressed as µmol/g dry wt/min were calculated for each time interval and were averaged for aerobic equilibration (aerobic) and postischemic (reperf) periods.

ff-IR; untreated early diabetic hearts (N=10), ff-IR/SEV; early diabetic hearts exposed to 2 vol.-% sevoflurane (N=10), and ff-IR/IL; early diabetic hearts treated with 1% Intralipid® at the onset of reperfusion (N=6). Data are expressed as mean ± SD and were analyzed by two-way repeated measures ANOVA.
