# Supplementary material for: Loss of Intralipid®- but Not Sevoflurane-Mediated Cardioprotection in Early Type-2 Diabetic Hearts of Fructose-Fed Rats: Importance of ROS Signaling
Source: PLoS One. 2014 Aug 15;9(8):e104971. doi: 10.1371/journal.pone.0104971 (PMC4134246; doi:10.1371/journal.pone.0104971)
Supplement: Figure S2 — Linoleoylcarnitine (C18∶2), oleoylcarnitine (C18;1), palmitoylcarnitine (C16∶0) levels as well as ratio between total tissue acylcarnitines (AC) and free carnitine in hearts from healthy (reproduced from reference 19 with permission) and fructose-fed rats subjected to 15 min ischemia and 3 min of reperfusion with/without 1% Intralipid® at the onset of reperfusion. (PDF) [file pone.0104971.s002.pdf]

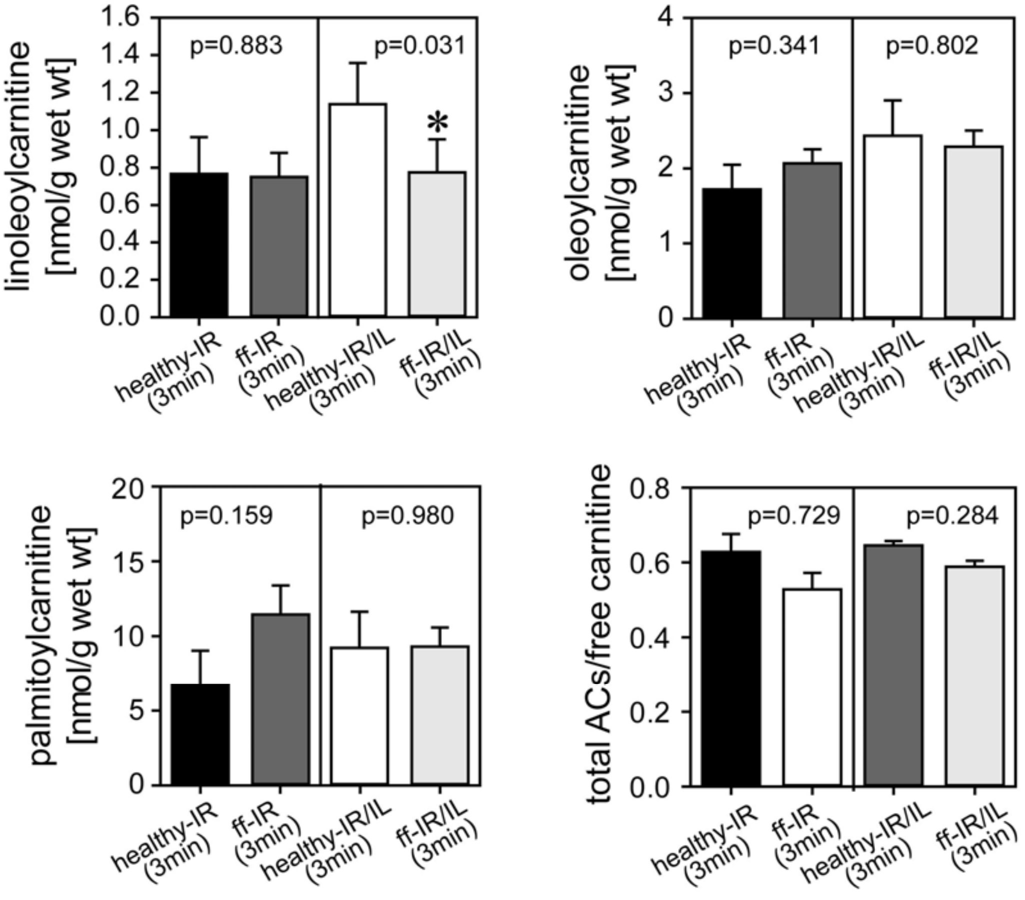

Figure S2: Linoleoylcarnitine (C18:2), oleoylcarnitine (C18;1), palmitoylcarnitine (C16:0) levels as well as ratio between total tissue acylcarnitines (AC) and free carnitine in hearts from healthy (reproduced from reference 19 with permission) and fructose-fed rats subjected to 15 min ischemia and 3 min of reperfusion with/without 1% Intralipid® at the onset of reperfusion.

healthy-IR (3 min), healthy hearts exposed to 15 min of ischemia and 3 min of reperfusion; healthy-IR/IL (3 min), healthy hearts exposed to 15 min of ischemia and 3 min of reperfusion and 1% Intralipid® at the onset of reperfusion; ff-IR (3 min), hearts from fructose-fed (ff) rats exposed to 15 min of ischemia and 3 min of reperfusion; ff-IR/IL (3 min), diabetic hearts exposed to 15 min of ischemia and 3 min of reperfusion and 1% Intralipid® at the onset of reperfusion.

*, significantly different from the corresponding healthy group. Data are mean (SEM). N=5 hearts in each group.
