# Supplementary material for: Loss of Intralipid®- but Not Sevoflurane-Mediated Cardioprotection in Early Type-2 Diabetic Hearts of Fructose-Fed Rats: Importance of ROS Signaling
Source: PLoS One. 2014 Aug 15;9(8):e104971. doi: 10.1371/journal.pone.0104971 (PMC4134246; doi:10.1371/journal.pone.0104971)
Supplement: Figure S3 — Linoleoylcarnitine (C18∶2), oleoylcarnitine (C18;1), palmitoylcarnitine (C16∶0) levels as well as ratio between total tissue acylcarnitines (AC) and free carnitine in hearts from healthy and fructose-fed rats aerobically perfused with/without 1% Intralipid®. (PDF) [file pone.0104971.s003.pdf]

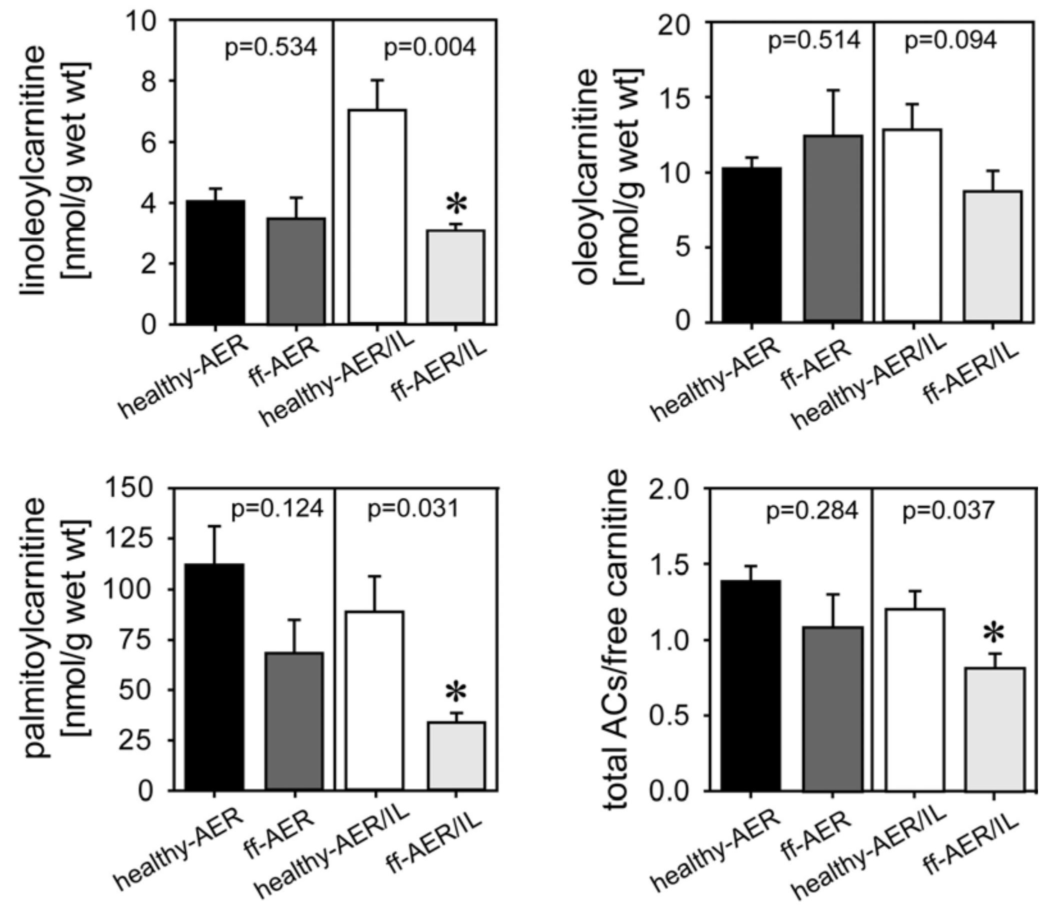

Figure S3: Linoleoylcarnitine (C18:2), oleoylcarnitine (C18;1), palmitoylcarnitine (C16:0) levels as well as ratio between total tissue acylcarnitines (AC) and free carnitine in hearts from healthy and fructose-fed rats aerobically perfused with/without 1% Intralipid®.

healthy-AER, time-matched aerobically perfused healthy hearts; ff-AER, time-matched aerobically perfused hearts from fructose-fed (ff) rats; healthy-AER/IL, time-matched aerobically perfused healthy hearts treated with 1% Intralipid® for 30 min; ff-AER/IL, time-matched aerobically perfused diabetic hearts treated with 1% Intralipid® for 30 min. *, significantly different from the corresponding healthy group. Data are mean (SEM). N=5-6 hearts in each group.
