# Supplementary material for: Loss of Intralipid®- but Not Sevoflurane-Mediated Cardioprotection in Early Type-2 Diabetic Hearts of Fructose-Fed Rats: Importance of ROS Signaling
Source: PLoS One. 2014 Aug 15;9(8):e104971. doi: 10.1371/journal.pone.0104971 (PMC4134246; doi:10.1371/journal.pone.0104971)
Supplement: Figure S4 — KCN titration experiments of cytochrome c oxidase activity. (PDF) [file pone.0104971.s004.pdf]

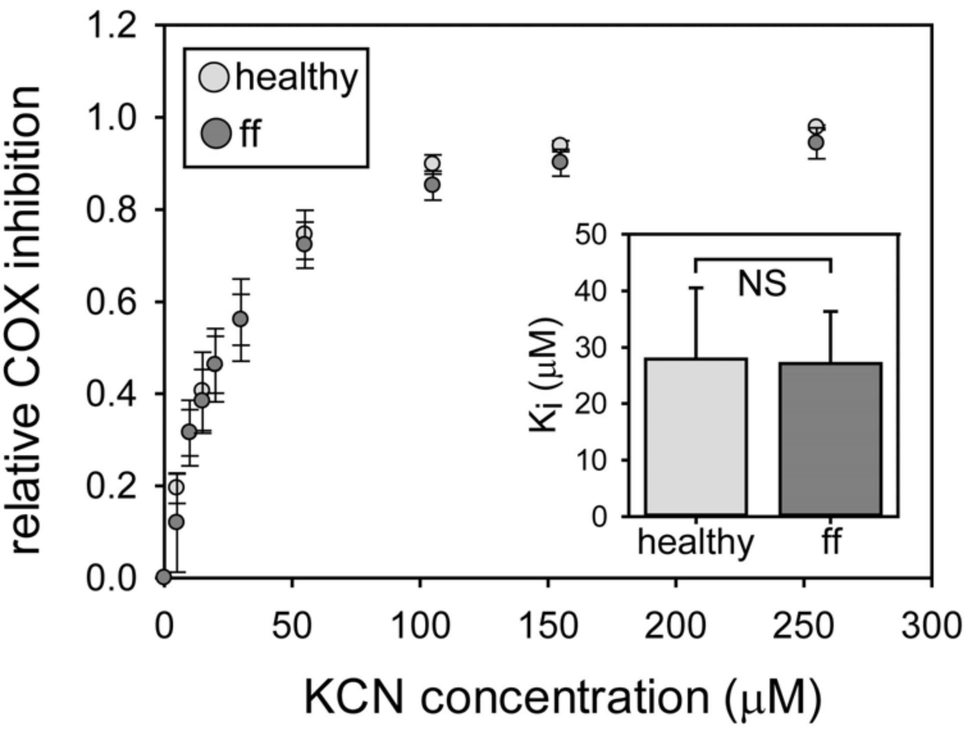

Figure S4: KCN titration experiments of cytochrome c oxidase activity.

To assess if cytochrome c oxidase (COX) responds differently to cyanide inhibition in cardiac mitochondria from fructose-fed rats as compared to healthy rats, we performed KCN titration experiments of cytochrome c oxidase activity in permeabilized cardiac fibers energized with complex IV substrates ascorbate (2 mM)/tetramethylphenylenediamine dihydrochloride (TMPD; 0.5 mM) to establish the concentration-dependent inhibition of complex IV. KCN induced a similar concentration-dependent inhibition of complex IV activity in fibers from fructose-fed rats as compared to healthy rats. The inhibition constant K_i of COX for cyanide was not affected by the dietary intervention (N=6).
