# Supplementary material for: Loss of Intralipid®- but Not Sevoflurane-Mediated Cardioprotection in Early Type-2 Diabetic Hearts of Fructose-Fed Rats: Importance of ROS Signaling
Source: PLoS One. 2014 Aug 15;9(8):e104971. doi: 10.1371/journal.pone.0104971 (PMC4134246; doi:10.1371/journal.pone.0104971)
Supplement: Figure S5 — Isoform 2 of complex IV and uncoupling protein 3 protein levels in healthy and diabetic hearts normalized to adenine nucleotide translocase. (PDF) [file pone.0104971.s005.pdf]

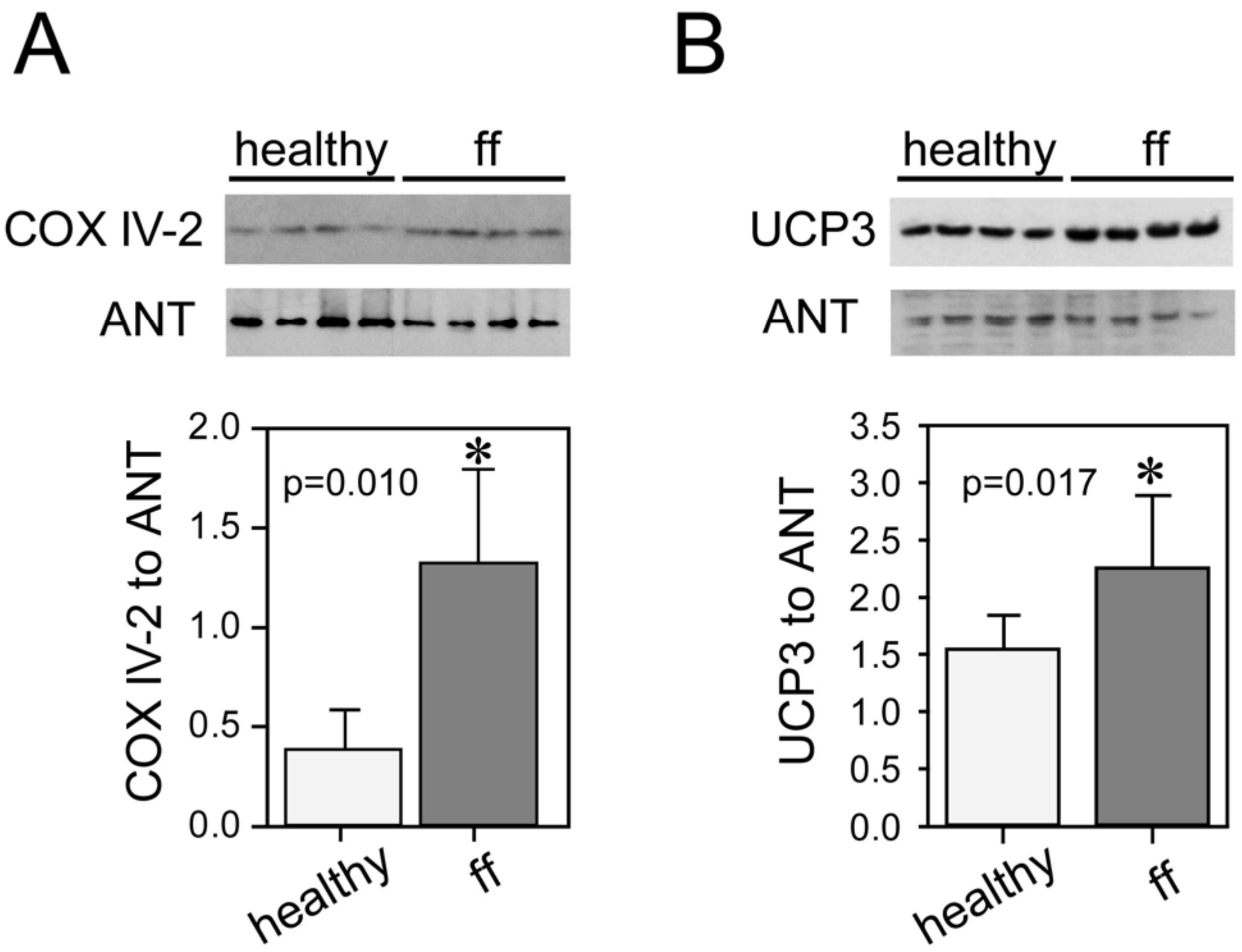

Figure S5: Panel A: increase in isoform 2 of complex IV (COX) subunit IV (COX IV-2) in cardiac mitochondria from fructose-fed (ff) rats as compared to healthy rats. Panel B: uncoupling protein 3 (UCP 3) is increased in cardiac mitochondria of fructose-fed (ff) rats as compared with healthy rats. ANT, adenine nucleotide translocase. *, significantly increased from healthy. Data are mean ± SD. N=4-5 hearts in each group.
