# Supplementary material for: Loss of Intralipid®- but Not Sevoflurane-Mediated Cardioprotection in Early Type-2 Diabetic Hearts of Fructose-Fed Rats: Importance of ROS Signaling
Source: PLoS One. 2014 Aug 15;9(8):e104971. doi: 10.1371/journal.pone.0104971 (PMC4134246; doi:10.1371/journal.pone.0104971)
Supplement: Table S1 — Hemodynamic data (full protocols; 30 min reperfusion time). (PDF) [file pone.0104971.s006.pdf]

Table S1. Hemodynamic data (full protocols; 30 min reperfusion time)

		**ff-IR**	**ff-IR/IL**	**ff-IR/SEV**	**ff-IR/SEV+MPG**	**ff-IR/MPG**
LVW [mmHg•L/min]	aerobic	8.53 (1.06)	8.37 (0.73)	8.09 (1.12)	8.06 (1.50)	8.17 (0.73)
	reperfusion	1.24 (1.27)	0.60 (0.75)	5.41 (0.90)*	1.77 (0.58)	2.71 (1.20)#
RM-ANOVA p(group); p(time); p(interaction)		**<0.001**; **<0.001**; **<0.001**				
DP [mmHg]	aerobic	63 (6)	59 (3)	59 (4)	64 (3)	58 (3)
	reperfusion	66 (9)	54 (8)**	65 (5)	64 (5)	59 (14)
RM-ANOVA p(group); p(time); p(interaction)		**0.034**; 0.569; 0.279				
PSP [mmHg]	aerobic	134 (7)	129 (6)	132 (8)	128 (10)	131 (9)
	reperfusion	83 (21)	65 (19)§	116 (7)†	85 (7)	103 (11)†
RM-ANOVA p(group); p(time); p(interaction)		**<0.001**; **<0.001**; **<0.001**				
CF [mL/min]	aerobic	20.5 (3.5)	21.3 (3.3)	20.0 (2.4)	21.6 (1.0)	19.8 (2.2)
	reperfusion	11.4 (7.4) #	7.1 (3.0)§	22.7 (3.2)$#	19.8 (3.2) #	17.7 (1.7) #
RM-ANOVA p(group); p(time); p(interaction)		**<0.001**; **<0.001**; **<0.001**				

Data are presented as mean (SD). Two-way repeated-measures analysis of variance (RM-ANOVA) followed by multiple comparison procedures (Holm-Sidak method, as appropriate) was used to compare groups. Significant group-time interactions are indicated in boldface. *, significantly increased compared to all other groups; #, significantly increased compared to ff-IR/IL; **, significantly decreased compared to ff-IR and ff-IR/SEV; †, significantly increased compared to ff-IR, ff-IR/IL, and ff-IR/SEV+MPG; §, significantly decreased compared to ff-IR; $, significantly increased compared to ff-IR, ff-IR/IL.

Abbreviations: LVW, left ventricular work; DP, diastolic pressure; PSP, peak systolic pressure; CF, coronary flow; ff-IR, hearts from fructose-fed rats exposed to ischemia-reperfusion (IR) without treatment (N=10); ff-IR/SEV, hearts from fructose-fed rats exposed to IR with sevoflurane (2 vol.-%) conditioning (N=10); ff-IR+IL, hearts from fructose-fed rats exposed to IR with Intralipid (1%) treatment at the onset of reperfusion; (N=6); ff-IR/SEV+MPG, hearts from fructose-fed rats exposed to IR with sevoflurane (2 vol.-%) and 10 μM N-(2-mercaptopropionyl)-glycine (N=4); ff-IR/MPG, hearts from fructose-fed rats exposed to IR with 10 μM N-(2-mercaptopropionyl)-glycine (N=4).
