# Supplementary material for: Loss of Intralipid®- but Not Sevoflurane-Mediated Cardioprotection in Early Type-2 Diabetic Hearts of Fructose-Fed Rats: Importance of ROS Signaling
Source: PLoS One. 2014 Aug 15;9(8):e104971. doi: 10.1371/journal.pone.0104971 (PMC4134246; doi:10.1371/journal.pone.0104971)
Supplement: Table S2 — Assessment of mitochondrial respiratory chain function in cardiac fibers from diabetic animals exposed to 2 vol.-% sevoflurane in the oxygraph chamber. (PDF) [file pone.0104971.s007.pdf]

Table S2. Assessment of mitochondrial respiratory chain function in cardiac fibers from diabetic animals exposed to 2 vol.-% sevoflurane in the oxygraph chamber

Oxygen consumption in presence of ADP		***Results***		
**Substrate/electron donor**	**Site(s) of electron input**	**ff-DMSO**	**ff-DMSO/SEV**	**p-value**
pyruvate/malate	Complex I (NADH:ubiquinone oxidoreductase)	0.93 (0.07)	0.80 (0.06)	**0.005**
succinate	Complex II (succinate:ubiquinone oxidoreductase)	0.97 (0.05)	0.93 (0.09)	0.371
decylubiquinol	Complex III (ubiquinol:ferricytochrome c oxidoreductase)	1.00 (0.16)	1.01 (0.18)	0.903
ascorbate/TMPD	Complex IV (cytochrome c oxidase)	1.02 (0.05)	1.01 (0.13)	0.927
Respiratory control ratio				
pyruvate/malate	Complex I (NADH:ubiquinone oxidoreductase)	7.0 (1.8)	5.8 (1.5)	0.216
succinate	Complex II (succinate:ubiquinone oxidoreductase)	2.9 (0.2)	2.7 (0.2)	**0.036**
decylubiquinol	Complex III (ubiquinol:ferricytochrome c oxidoreductase)	2.8 (1.5)	2.4 (0.9)	0.555

Complex I, complex II, and complex IV functions were measured polarographically by monitoring oxygen consumption in the presence of specific substrates and ADP in saponin-skinned cardiac fibers from fructose-fed rats using high-resolution respirometry. The measured oxygen consumption is normalized to oxygen consumption in the absence of sevoflurane/DMSO and is thus a dimensionless quantity. Data are presented as mean (SD). N=4-6.

Abbreviations:

DMSO, dimethyl sulfoxide (0.1%) used as solvent for sevoflurane; DMSO/SEV, sevoflurane (2-vol% dissolved in DMSO); ff, fructose fed; TMPD, tetramethyl-p-phenylene diamine, an artificial electron carrier which is reduced by ascorbate producing electrons that are transferred to cytochrome c.
