# Supplementary material for: Loss of Intralipid®- but Not Sevoflurane-Mediated Cardioprotection in Early Type-2 Diabetic Hearts of Fructose-Fed Rats: Importance of ROS Signaling
Source: PLoS One. 2014 Aug 15;9(8):e104971. doi: 10.1371/journal.pone.0104971 (PMC4134246; doi:10.1371/journal.pone.0104971)
Supplement: Table S3 — Assessment of mitochondrial respiratory chain function in hearts from diabetic rats aerobically perfused with 1% Intralipid®. (PDF) [file pone.0104971.s008.pdf]

Table S3. Assessment of mitochondrial respiratory chain function in hearts from diabetic rats aerobically perfused with 1% Intralipid®

Oxygen consumption in presence of ADP		***Results***		
**Substrate/electron donor**	**Site(s) of electron input**	**ff-AER**	**ff-AER/IL**	**p-value**
pyruvate/malate	Complex I (NADH:ubiquinone oxidoreductase)	10.3 (2.9)	14.3 (3.0)	**0.018**
pyruvate/malate/succinate	Complex I+II	13.8 (4.2)	17.4 (3.8)	0.111
succinate	Complex II (succinate:ubiquinone oxidoreductase)	8.3 (1.9)	10.8 (2.3)	**0.047**
ascorbate/TMPD	Complex IV (cytochrome c oxidase)	21.0 (5.3)	21.8 (3.0)	0.763
palmitoylcarnitine/malate	Complex I+Electron transfer flavoprotein-ubiquinone oxidoreductase	2.7 (0.9)	4.5 (0.8)	**0.004**
Oxygen consumption in absence of ADP (mitochondrial leak)				
pyruvate/malate	Complex I (NADH:ubiquinone oxidoreductase)	2.3 (1.1)	1.8 (0.5)	0.271
succinate	Complex II (succinate:ubiquinone oxidoreductase)	4.3 (1.1)	5.6 (1.3)	0.071
palmitoylcarnitine/malate	Complex I+Electron transfer flavoprotein-ubiquinone oxidoreductase	1.2 (1.2,1.6)	1.4 (1.1,2.2)	0.852

Respiratory control ratio				
pyruvate/malate	Complex I (NADH:ubiquinone oxidoreductase)	4.5 (1.3)	7.0 (1.6)	**0.007**
succinate	Complex II (succinate:ubiquinone oxidoreductase)	1.8 (0.3)	2.7 (0.4)	**0.002**
palmitoylcarnitine/malate	Complex I+Electron transfer flavoprotein-ubiquinone oxidoreductase	2.0 (0.6)	2.5 (0.6)	0.087

Rat hearts were aerobically perfused with/without 1% Intralipid®. Complex I, complex II, and complex IV functions were measured polarographically by monitoring oxygen consumption in presence of specific substrates and ADP in saponin-skinned cardiac fibers using high-resolution respirometry. The measured oxygen consumption (normalized to citrate synthase activity) is expressed as nmol O_2*s^{-1}/CS. Complex III activity, measured spectrophotometrically using the MitoTox™ OXPHOS Complex III Activity Kit (MT-OX3, Abcam Inc. Toronto (ON), Canada), was unaffected by Intralipid®. Data are presented as mean (SD) or median (25th percentile, 75th percentile). N=6-10 in all groups.

Abbreviations:

ff-AER, aerobically perfused hearts from fructose-fed animals without treatment; ff-AER/IL, aerobically perfused hearts from fructose-fed animals exposed to 1% Intralipid®; TMPD, tetramethyl-p-phenylene diamine, an artificial electron carrier which is reduced by ascorbate producing electrons that are transferred to cytochrome c
